# Healing condition of WATCHMAN surface 2.5 years after implantation observed in cardiac surgery

**DOI:** 10.1093/ehjcr/ytae198

**Published:** 2024-04-20

**Authors:** Masamichi Yano, Yasuyuki Egami, Haruhiko Kondoh, Masami Nishino

**Affiliations:** Division of Cardiology, Osaka Rosai Hospital, 3-1179 Nagasonecho, kita-ku, Sakai, Osaka 591-8025, Japan; Division of Cardiology, Osaka Rosai Hospital, 3-1179 Nagasonecho, kita-ku, Sakai, Osaka 591-8025, Japan; Division of Cardiovascular Surgery, Osaka Rosai Hospital, 1179-3 Nagasonecho, kita-ku, Sakai, Osaka 591-8025, Japan; Division of Cardiology, Osaka Rosai Hospital, 3-1179 Nagasonecho, kita-ku, Sakai, Osaka 591-8025, Japan

A 74-year-old female with paroxysmal atrial fibrillation underwent successful left atrial (LA) appendage closure (LAAC) using a 27 mm WATCHMAN device (Boston Scientific, Marlborough, MA, USA), chosen based on a maximal ostial diameter of 22.0 mm measured by transoesophageal echocardiography (TEE) (see Supplementary material online, *Supplementary Video S1*). She was prescribed a combination of aspirin and rivaroxaban for 45 days post-LAAC, followed by dual antiplatelet therapy with aspirin and clopidogrel for six months. Aspirin monotherapy commenced thereafter. At the 45-day follow-up, TEE revealed no evidence of thrombus formation or peri-device leakage; however, endothelialization of the device surface was indeterminate (see Supplementary material online, *Supplementary Videos S2* and *S3*). Concurrently, mitral regurgitation progressed to a severe condition and surgical intervention was considered. Two months prior to surgery, TEE indicated partial endothelial coverage at the device’s superior edge but not on the mitral annulus side (*Figure 1A* and *B* and Supplementary material online, *Video S1*). Mitral valve repair was electively performed 934 days post-LAAC. Intraoperative findings showed white and reddish-brown tissue enveloping the superior edge of the device, with the mitral annulus side remaining exposed (*Figure 1C* and *D* and Supplementary material online, *Video S2*). Post-operatively, warfarin was administered for three months, with aspirin reintroduced thereafter. A computed tomography at 10 months post-surgery displayed contrast enhancement within the left atrial appendage, suggesting incomplete device endothelialization (see Supplementary material online, *Figure S1*). The endothelialization timeline for WATCHMAN device is not fully understood, with various reports depicting the state of endothelialization post-deployment.^1–3^ Our case exhibited delayed endothelialization, persisting 2.5 years following device.

**Figure 1:**
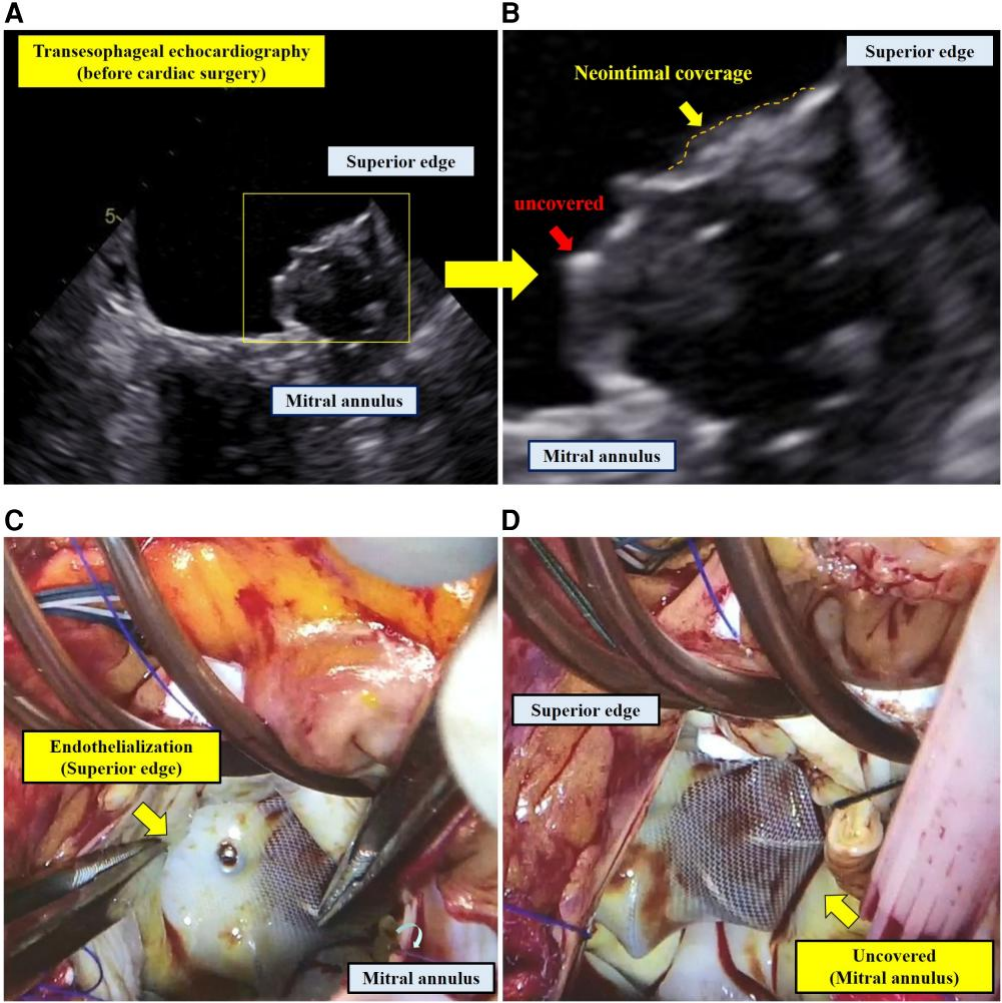
Transoesophageal echocardiography at 2 months before surgery. (*A*) It showed that the intimal coverage was confirmed in the surface in the superior edge of WATCHMAN device, while the device remained uncovered in the mitral annulus side. (*B*) The white and reddish-brown tissue coverage was observed at the superior edge of WATCHMAN device. (*C*) Approximately half of the device remained uncovered in particular at the mitral annulus side.

## Supplementary Material

ytae198_Supplementary_Data

## Data Availability

The datasets analysed in this study are available from the corresponding author upon reasonable request.
