# Supplementary material for: Healing condition of WATCHMAN surface 2.5 years after implantation observed in cardiac surgery
Source: Eur Heart J Case Rep. 2024 Apr 20;8(4):ytae198. doi: 10.1093/ehjcr/ytae198 (PMC11060099; doi:10.1093/ehjcr/ytae198)
Supplement: ytae198_Supplementary_Data [file ytae198_supplementary_data.zip › Supplementary Figure legends.docx]

**Supplementary Figure legends**

**Supplementary Figure 1**

A computed tomography 10 months post-surgery. Contrast enhancement within the left atrial appendage (yellow arrow)

LAA, left atrial appendage
