# Supplementary material for: Healing condition of WATCHMAN surface 2.5 years after implantation observed in cardiac surgery
Source: Eur Heart J Case Rep. 2024 Apr 20;8(4):ytae198. doi: 10.1093/ehjcr/ytae198 (PMC11060099; doi:10.1093/ehjcr/ytae198)
Supplement: ytae198_Supplementary_Data [file ytae198_supplementary_data.zip › Supplementary Video legends.docx]

**Supplementary Video legends**

**Supplementary Video 1**

The angiography after LAAC with a 27 mm WATCHMAN

**Supplementary Video 2**

TEE (Doppler at 45 ^o^ view) at 45 days after the device placement.

**Supplementary Video 3**

TEE (3D and at 45 ^o^/135 ^o^ view) at 45 days after the device placement.

**References**

1. Reddy VY, Doshi SK, Kar S, Gibson DN, Price MJ, Huber K, Horton RP, Buchbinder M, Neuzil P, Gordon NT, Holmes DR, Jr. 5-Year Outcomes After Left Atrial Appendage Closure: From the PREVAIL and PROTECT AF Trials. J Am Coll Cardiol 2017;70:2964-2975.

2. Saw J, Fahmy P, DeJong P, Lempereur M, Spencer R, Tsang M, Gin K, Jue J, Mayo J, McLaughlin P, Nicolaou S. Cardiac CT angiography for device surveillance after endovascular left atrial appendage closure. Eur Heart J Cardiovasc Imaging 2015;16:1198-1206.

3. Holmes DR, Reddy VY, Turi ZG, Doshi SK, Sievert H, Buchbinder M, Mullin CM, Sick P. Percutaneous closure of the left atrial appendage versus warfarin therapy for prevention of stroke in patients with atrial fibrillation: a randomised non-inferiority trial. Lancet 2009;374:534-542.

4. Kar S, Hou D, Jones R, Werner D, Swanson L, Tischler B, Stein K, Huibregtse B, Ladich E, Kutys R, Virmani R. Impact of Watchman and Amplatzer devices on left atrial appendage adjacent structures and healing response in a canine model. JACC Cardiovasc interv 2014;7:801-809.

5. Nakase M, Asami M, Yahagi K, Sato Y, Nakayama A, Ninomiya K, Tanaka T, Horiuchi Y, Yuzawa H, Komiyama K, Tanaka J, Aoki J, Virmani R, Mori M, Tanabe K. Autopsy findings of left atrial appendage closure device. Cardiovasc Pathol 2022;56:107384.

6. Sick PB, Schuler G, Hauptmann KE, Grube E, Yakubov S, Turi ZG, Mishkel G, Almany S, Holmes DR. Initial worldwide experience with the WATCHMAN left atrial appendage system for stroke prevention in atrial fibrillation. J Am Coll Cardiol 2007;49:1490-1495.

7. Schwartz RS, Holmes DR, Van Tassel RA, Hauser R, Henry TD, Mooney M, Matthews R, Doshi S, Jones RM, Virmani R. Left atrial appendage obliteration: mechanisms of healing and intracardiac integration. JACC Cardiovasc interv　2010;3:870-877.

8. Massarenti L, Yilmaz A. Incomplete endothelialization of left atrial appendage occlusion device 10 months after implantation. J Cardiovasc Electrophysiol 2012;23:1384-1385.

9. Cresti A, Galli CA, Alimento ML, De Sensi F, Baratta P, D'Aiello I, Limbruno U, Pepi M, Fusini L, Maltagliati AC. Does mitral regurgitation reduce the risks of thrombosis in atrial fibrillation and flutter? J Cardiovasc Med 2019;20:660-666.

10. Kranidis A, Koulouris S, Filippatos G, Kappos K, Tsilias K, Karvounis H, Exadaktylos N. Mitral regurgitation protects from left atrial thrombogenesis in patients with mitral valve disease and atrial fibrillation. Pacing Clin Electrophysiol 2000;23:1863-1866.

11. Van Laer SL, Verreyen S, Winkler KM, Miljoen H, Sarkozy A, Heuten H, Saenen J, Van Herck P, Van de Heyning CM, Heidbuchel H, Claeys MJ. Effect of Mitral Regurgitation on Thrombotic Risk in Patients With Nonrheumatic Atrial Fibrillation: A New CHA(2)DS(2)-VASc Score Risk Modifier? Am J Cardiol 2021;145:69-76.

12. Saw J, Nielsen-Kudsk JE, Bergmann M, Daniels MJ, Tzikas A, Reisman M, Rana BS. Antithrombotic Therapy and Device-Related Thrombosis Following Endovascular Left Atrial Appendage Closure. JACC Cardiovasc interv 2019;12:1067-1076.

**Video legends**

**Figure legends**

**Figure 1**

1. The angiographic imaging of LAAC with a 27 mm WATCHMAN placement with a complete LAA seal and optimal device position.
2. Transesophageal echocardiography imaging at 45 days after the device deployment. It showed no thrombus along the device and no peri-device leak, and the endothelialization on the device surface could not clearly confirmed.

LAA=left atrial appendage; LAAC=left atrial appendage closure

**Figure 2**

Transesophageal echocardiography at 2 months before surgery. It showed that the intimal coverage was confirmed in the surface in the superior edge of WATCHMAN device (yellow arrow), while the device remained uncovered in the mitral annulus side (red arrow). The protrusion of the device toward the left atrium was observed in particular at the mitral annulus side (green arrow)

**Figure 3**

1. The white and reddish-brown tissue coverage was observed at the superior edge of WATCHMAN device.
2. Approximately half of the device remained uncovered in particular at the mitral annulus side.
