# Supplementary figures and images for: Healing condition of WATCHMAN surface 2.5 years after implantation observed in cardiac surgery
Source: Eur Heart J Case Rep. 2024 Apr 20;8(4):ytae198. doi: 10.1093/ehjcr/ytae198 (PMC11060099; doi:10.1093/ehjcr/ytae198)

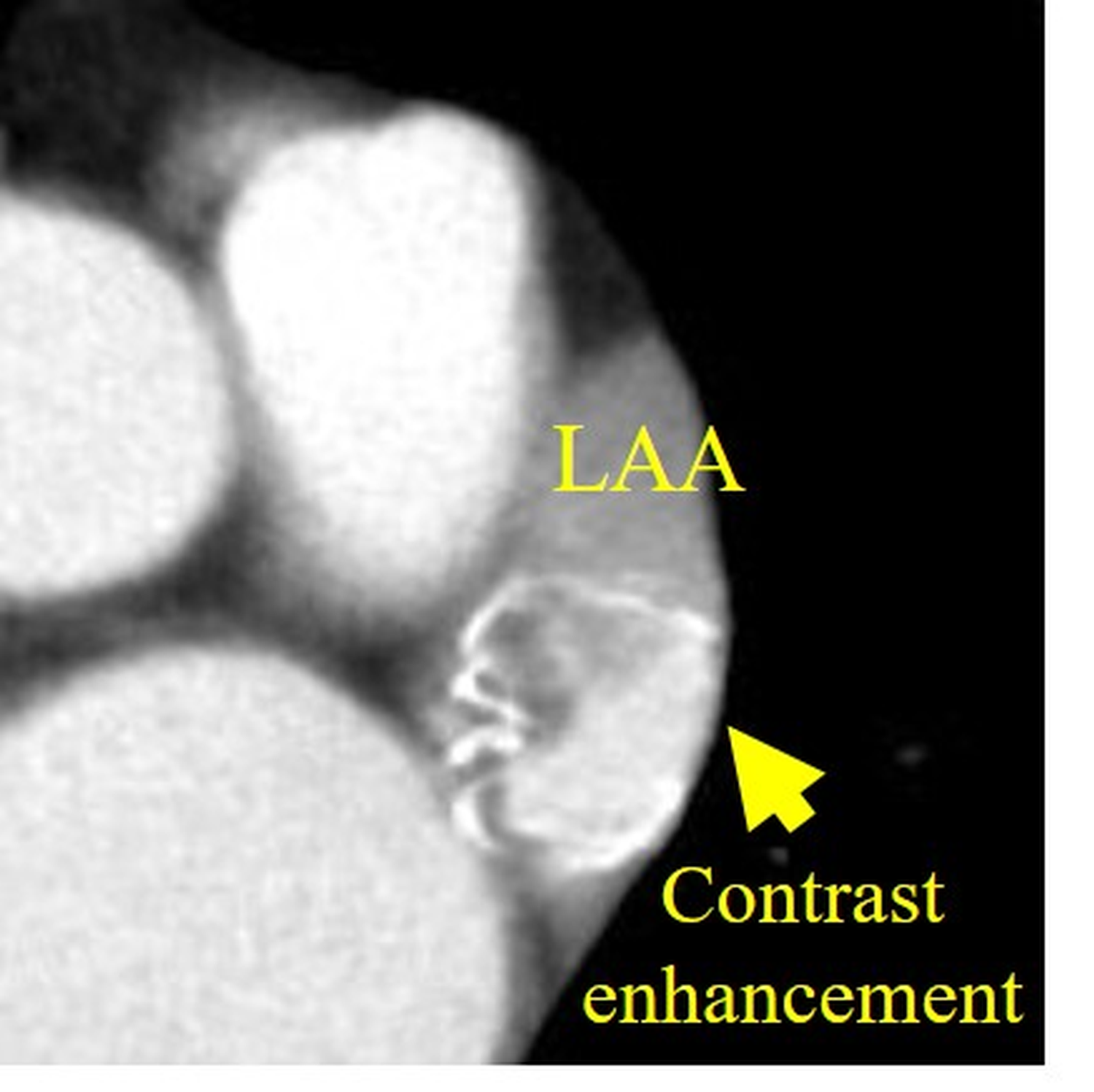

Supplement: ytae198_Supplementary_Data [file ytae198_supplementary_data.zip › Supplementary Figure.tif]
